# Recent advances in stem cell therapeutics and tissue engineering strategies

**DOI:** 10.1186/s40824-018-0148-4

**Published:** 2018-12-19

**Authors:** Seong Gyu Kwon, Yang Woo Kwon, Tae Wook Lee, Gyu Tae Park, Jae Ho Kim

**Affiliations:** 1 Department of Physiology, Pusan National University School of Medicine, Yangsan, 50612 Gyeongsangnam-do Republic of Korea; 2 Research Institute of Convergence Biomedical Science and Technology, Pusan National University Yangsan Hospital, Yangsan, 50612 Republic of Korea

**Keywords:** Tissue injury, Nanoparticle, Stem cells, Biomaterials, Tissue engineering

## Abstract

**Background:**

Tissue regeneration includes delivering specific types of cells or cell products to injured tissues or organs for restoration of tissue and organ function. Stem cell therapy has drawn considerable attention since transplantation of stem cells can overcome the limitations of autologous transplantation of patient’s tissues; however, it is not perfect for treating diseases. To overcome the hurdles associated with stem cell therapy, tissue engineering techniques have been developed. Development of stem cell technology in combination with tissue engineering has opened new ways of producing engineered tissue substitutes. Several studies have shown that this combination of tissue engineering and stem cell technologies enhances cell viability, differentiation, and therapeutic efficacy of transplanted stem cells.

**Main body:**

Stem cells that can be used for tissue regeneration include mesenchymal stem cells, embryonic stem cells, and induced pluripotent stem cells. Transplantation of stem cells alone into injured tissues exhibited low therapeutic efficacy due to poor viability and diminished regenerative activity of transplanted cells. In this review, we will discuss the progress of biomedical engineering, including scaffolds, biomaterials, and tissue engineering techniques to overcome the low therapeutic efficacy of stem cells and to treat human diseases.

**Conclusion:**

The combination of stem cell and tissue engineering techniques overcomes the limitations of stem cells in therapy of human diseases, and presents a new path toward regeneration of injured tissues.

## Background

The growing tendency of increased life expectancy as well as increased incidence of age-related degenerative diseases and tissue damage requires the use of allogenic or autologous grafts for tissue repair. Although transplantation of tissues or cells is innovative and has been applied to a lot of treatments, its application in clinical settings is still limited [1]. Accumulating evidence suggests that stem cells can accelerate the tissue regeneration through various mechanisms. To date, a variety of stem cells, including mesenchymal, embryonic, and induced pluripotent stem cells, have been reported to promote regeneration of damaged tissues [2]. Although stem cell therapy provides a new paradigm in tissue regeneration, they have limitation in clinical application due to poor survival and differentiation potentials of the transplanted cells [3]. To overcome these limitations, tissue engineering technology has been used to improve the viability and proliferative capacity of stem cells. Tissue engineering is the use of a combination of cells, biomaterials, biochemical and physicochemical factors, and engineering technologies to improve or replace biological tissues [4]. In this paper, we will review the types of stem cells, their use in various tissues, and tissue regeneration through stem cell engineering. In addition, there are many other kinds of stem cells that can be used for tissue regeneration; however, in this review, we focus on the above-mentioned stem cells for tissue regeneration.

### Types of stem cells for tissue regeneration

Mesenchymal stem cells (MSCs) can be isolated from various tissues, such as adipose tissue, tonsil, and bone marrow. MSCs show plastic adherent properties under normal culture conditions and have a fibroblast-like morphology. They express specific cell surface markers including CD73, CD90, and CD105. MSCs have the potential for self-renewal and differentiation potential into mesodermal lineages, including adipocytes, muscles, chondrocytes, and osteoblasts [2]. In addition to the differentiation potential, increasing body of evidence suggests that MSCs possess immune modulatory function and pro-angiogenic activity which are beneficial for tissue regeneration [5]. MSCs interfere with dendritic cell and T-cell function and generate a local immunosuppressive environment by secreting various immune-modulatory cytokines [6]. Moreover, MSCs promote angiogenesis by secreting pro-angiogenic factors [7]. Therefore, MSC-based clinical trials have been conducted worldwide for various human diseases, including cardiovascular, bone and cartilage, neuronal, and inflammatory diseases [8]. Several MSC-based cell therapeutics are commercially available [9], although their therapeutic efficacy is still in debate.

Embryonic stem cells (ESCs) are pluripotent stem cells derived from the inner cell mass of blastocysts, and they can differentiate to specific cell types by controlling culture conditions [10]. Recently, clinical trials were initiated to test the safety and potential efficacy of human ESCs in several diseases, including spinal cord injury, macular degeneration, diabetes and heart diseases. In 2010, Geron Corporation transplanted hESC-derived oligodendrocyte precursors, GRNOPC1, into five patients with spinal cord injury, and the clinical trial data suggest long-term safety of the therapy as well as reduced spinal cord cavitation in four of the five patients [11]. In addition, Advanced Cell Technology (MA, USA) tested human ESC-derived retinal pigment epithelium for age-related macular degeneration and Stargardt disease, a juvenile form of macular degeneration, and the clinical trial data have shown positive safety data with no tumorigenicity and improved clinical data in some patients [12]. Although ESCs have prominent advantages such as pluripotency and self-renewal potential, there are several obstacles hindering the clinical application of ESC-based cell therapeutics [13]. Because ESCs are derived from an embryo, they are allogenic cells to the patient and thus can be subjected to immune rejection. [14]. Secondly, it is difficult to induce differentiation into a desired cell type with 100% efficiency, thus a small fraction of undifferentiated cells might remain and form teratomas. Moreover, there are ethical issues because human ESCs are derived from human embryo, which has delayed clinical application of ESCs.

These ESC-associated issues were alleviated by the work of Yamanaka and colleagues on somatic cell reprogramming [15]. They demonstrated that somatic cells could be reprogrammed to a primordial stem cell state by introducing four pluripotency-inducing transcription factors. Since induced pluripotent stem cells (iPSCs) could be reprogrammed from adult somatic cells, they are free from ethical concerns [16]. Although iPSCs do not negate the risk of generating tumors, transplantation of autologous iPSC-derived cell therapeutics could help solve the immunological problem associated with transplantation of ESC-derived cells [17]. Japan’s RIKEN Institute successfully transplanted the world’s first iPSC-derived therapy into age-related macular degeneration patients [18]. However, there is a risk of neoplastic development from cells differentiated from iPSCs, because reprogramming factors are associated with the development of tumors [19].

### Development of stem cell-activating growth factors and peptides

**Fig. 1:**
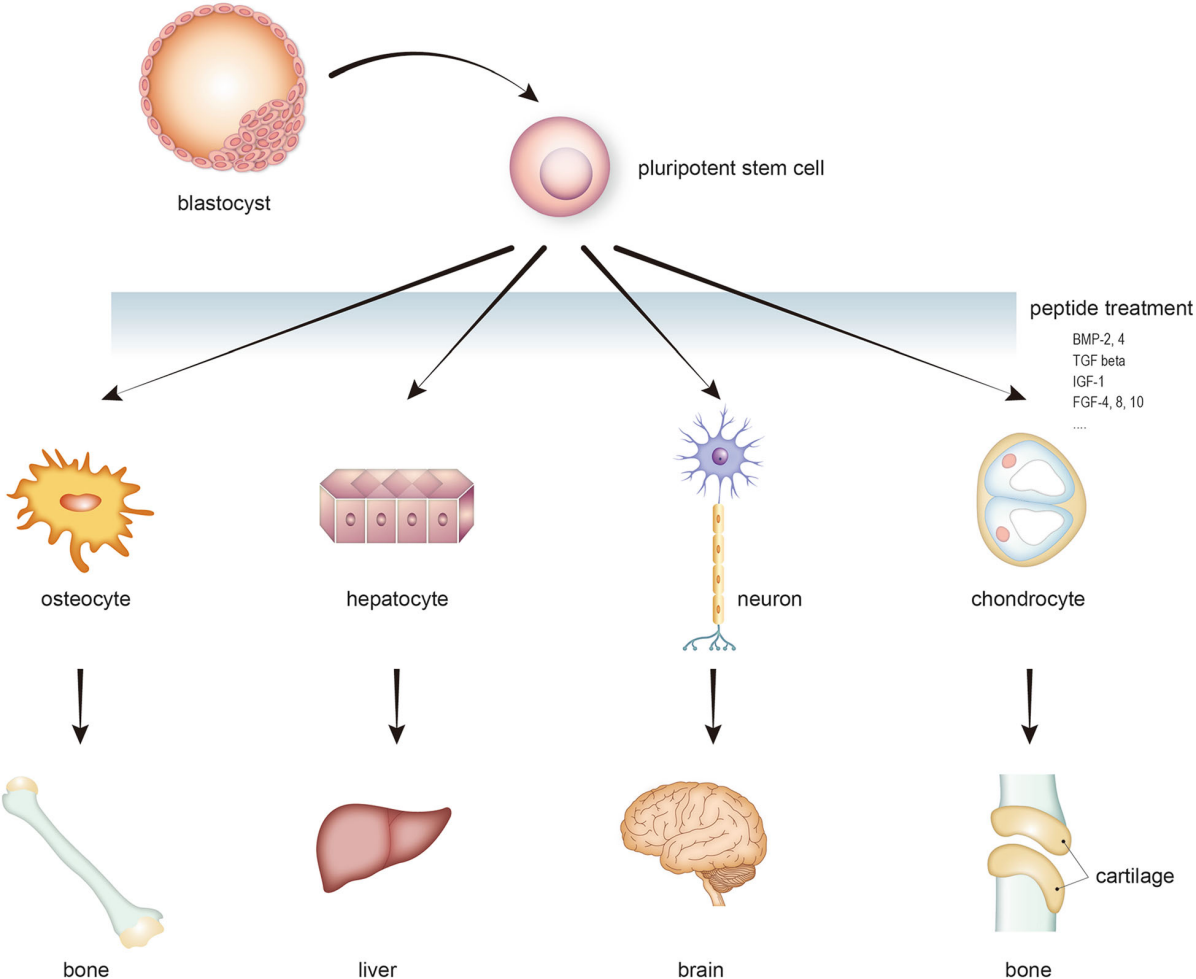
Stem cell differentiation in response to specific ligands or growth factors

Stem cells can differentiate into different kinds of cell types in response to specific ligands or growth factors (Fig. 1) [20]. Direct transplantation of stem cells into injured tissues was found to be effective in animal models; however, the possibility of inducing local ischemia or thrombosis has been raised [21]. Moreover, stem cell-based cell therapy has been hampered by poor survival of transplanted stem cells in vivo. Therefore, there is a need to develop stem cell-activating factors that enhance the survival, paracrine effects, and therapeutic efficacy of transplanted stem cells. In particular, BMPs have been shown to exert novel effects on cartilage and bone regeneration in several animal experiments. It has been reported that bone morphogenetic proteins (BMPs) and bone-forming peptide-3 stimulated differentiation of MSCs to osteoblasts [22, 23]. Among the various types of BMPs, both BMP2 and BMP7 have been shown to play important roles in bone and cartilage regeneration [24, 25].

Not only growth factors but also extracellular matrix proteins have been shown to promote the regenerative potentials of stem cells. Co-transplantation of MSCs along with collagen matrix or fibrin to the injured tissue site is now widely used clinically [26]. Periostin, an extracellular matrix protein that is expressed in the periosteum and periodontal ligaments, has been identified as a secreted protein of MSCs. Recombinant periostin protein stimulates proliferation, adhesion, and survival of MSCs in vitro, and co-implantation of MSCs and recombinant periostin protein significantly accelerates bone regeneration by increasing angiogenesis in a calvarial defect animal model [27]. Moreover, recombinant periostin and its fasciclin I domain promote therapeutic angiogenesis in a murine model of chronic limb ischemia [28]. Periostin stimulates angiogenesis and chemotaxis of endothelial colony forming cells through a mechanism involving β3 and β5 integrins. Recently, a short peptide sequence (amino acids 142–151), which is responsible for periostin-mediated angiogenesis, has been identified by serial deletion mapping of the first fasciclin I domain [29]. These results suggest that periostin can be applied for cell therapy by stimulating the pro-angiogenic and tissue regenerative potentials of MSCs.

In addition, it has been reported that co-transplantation of N-acetylated proline-glycine-proline, a peptide produced by the degradation of collagen, accelerates repair of dermal wounds by stimulating migration and engraftment of transplanted endothelial colony forming cells [30]. These results demonstrate that pro-angiogenic peptides, including periostin and N-acetylated proline-glycine-proline, promote regenerative potentials of transplanted stem cells by accelerating angiogenesis.

### Stem cells engineered with nanomaterials

While growth factors and cytokines can affect the biological functions of stem cells from “outside”, there are several ways to manipulate them from “inside”, as an approach on a more fundamental level. Gene therapy using viral expression systems is a well-known traditional method for manipulating the biological functions of stem cells from “inside”. However, viral expression systems have been reported to induce immune and inflammatory reactions in host tissues, and genetic mutations in host DNA can occur [31]. Therefore, development of highly efficient non-viral expression system is important for stem cell research. For instance, reprogramming or direct conversion of somatic cells by using non-viral gene expression system have great potential for clinical application of the reprogramming cells. Replacing viruses with alternative extracellular chemicals or delivery systems can reduce tumor formation. Non-viral methods include electroporation of cell membrane or delivery of genes in a form complexed with liposome or cationic polymers. Several types of nanoparticles have been developed for non-viral delivery of reprogramming factors into cells. These nanoparticles are composed of mesoporous silica, calcium phosphate, chitosan, cationic polymers, and magnetic nanoparticles [32]. Recently, graphene oxide-polyethylenimine complexes have been reported to be an efficient and safe system for mRNA delivery for direct reprogramming of somatic cells to induced neurons [33]. Therefore, improvement of gene delivery efficiency using nanoparticles will be highly useful for direct conversion or reprogramming of somatic cells.

## Biomaterials enhancing the therapeutic efficacy of stem cells

Tissues are composed of two components: cells and their surrounding extracellular matrix (ECM), which is known to play an important role in cell proliferation and differentiation. The main function of the ECM is maintaining cell growth and supplying essential components to cells [34]. ECM has been reported to create a framework for cell growth and to efficiently provide the nutrients or growth factors needed for cells [35]. It is difficult to naturally repair a large-size tissue defect by supplying cells to the injured sites, since not only the cells, but also the ECM are lost. Therefore, to promote tissue regeneration, it is necessary to make an artificial ECM environment for transplanted cells, and biomaterials are useful substitutes for ECM, and are also useful in cell therapy. The biomaterial scaffold should be porous for infiltration by cells into scaffolds, and for the supply of oxygen and nutrients to cells. In addition, the scaffold should be biodegradable for proper replacement of damaged tissues with the transplanted cells [36].

**Fig. 2:**
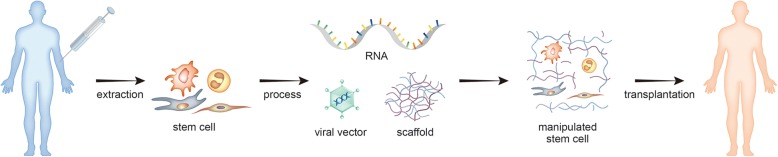
Stem cell engineering strategy

In terms of biomaterials, a variety of synthetic and natural materials have been developed. In particular, biodegradable polymers, such as collagen, gelatin, fibrin, hyaluronic acid, and poly(lactic-co-glycolic acid), are highly useful for tissue engineering [37]. The combination of these scaffolds and stem cells was used for skin wound healing [38]. The osteogenic efficiency of MSCs was confirmed in duck’s foot-derived collagen/hydroxyapatite scaffolds [39]. In addition, the increase of chondrogenic differentiation of MSCs in 3D alginate hydrogels was experimentally confirmed [40]. Neural stem cells have been used for treatment of neurodegenerative disease or stroke in pre-clinical and clinical studies; however, differentiation of neural stem cells to functional neurons, reconnection with host neural cells, and correct transmission of nerve signals are still obstacles to overcome [41]. Therefore, to enhance the survival and differentiation potentials of transplanted stem cells, it is necessary to combine biomaterials with growth factors, cytokines, and cell adhesive substances (Fig. 2).

### 3D bioprinting for tissue engineering

**Fig. 3:**
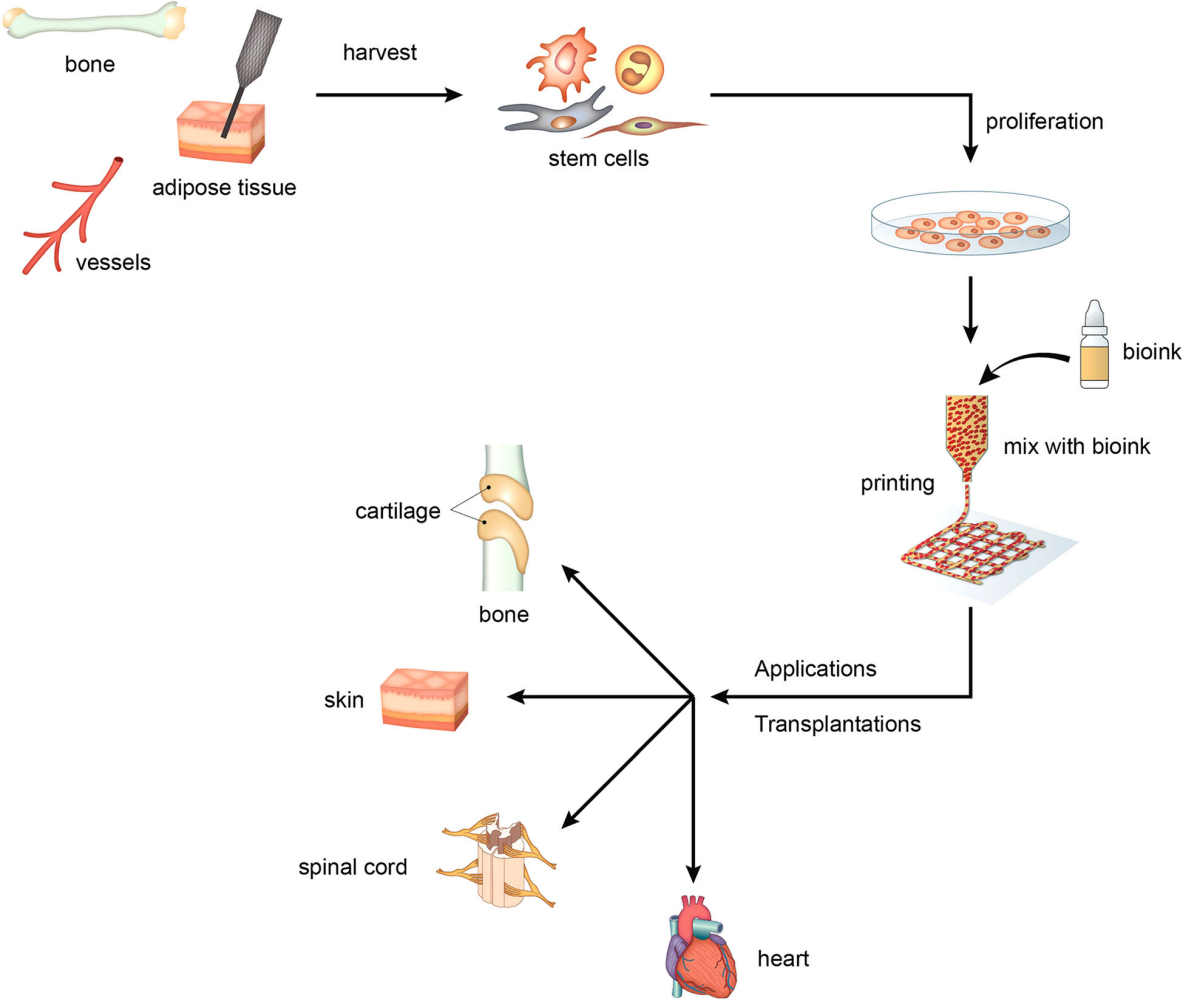
3D bioprinting of stem cells

Biomaterial scaffolds can be used as structural components for different parts of tissues, such as blood vessels, skin, and corneal tissues [42, 43]. Making 3D scaffolds and culturing stem cells on them improves the regenerative activity of stem cells for damaged bone and cartilage. Most tissues are composed of different cell types and multi-layered structures. Therefore, multi-layered 3D scaffolds are needed for construction of engineered tissues using stem cells. Currently, 3D bioprinting has drawn attention in the field of biotechnology for producing multi-layered structure. Since the first technology for 3D bioprinting cells had been reported, there have been great advances in 3D bioprinting-based tissue engineering [44]. Using 3D bioprinting, various cell types can be positioned in specific locations in multi-layered structures for constructing different tissues or organs (Fig. 3) [45]. Bioprinting technologies include inkjet [46] and laser deposition [47].

In using inkjet printer technology, however, since the cells are printed in the same manner as a commercial printer, various problems arise. For example, in order to print stem cells through an inkjet printer, the material that is added to the cells must be in a liquid form and, subsequently, have a 3D structure after injection [48]. However, employing crosslinking agents to form 3D structures can impair cellular viability [49]. Despite these drawbacks, remarkable advances have been made due to the advantage of 3D printing cells being possible with slight modifications to commercial inkjet printers on the market [50–54]. Just as laser printers have become popular, laser printers for 3D bioprinting have also been developed. Unlike inkjet printers, laser printers do not apply physical stresses and do not require additives to maintain a liquid form. The viability of cells is higher than 95% after being printed, and apoptosis and cell proliferation are not affected [55].

For 3D bioprinting, bioinks are needed for printing of stem cells into 3D structures, and hydrogels are widely used as bioinks. Each bioink has its own characteristics and is used for specific purposes [56]. Natural bioinks include alginate, gelatin, collagen I, and fibrin; synthetic bioinks include polyethylene glycol and pluronic gels [57]. These materials have chemical and physical properties appropriate for bioink, and they serve as scaffolds, similar to those of the ECM [58]. In order to mimic the ECM in vivo, de-cellularized extracellular matrix (dECM) scaffold has been developed. dECM is obtained by processing original tissues with chemicals, or using enzymatic methods to remove cellular components [59]. Therefore, dECM is highly useful for 3D bioprinting of stem cells, or their differentiated progeny cells.

In the regeneration of thick tissues, not only the regeneration of the tissue itself, but also the regeneration of blood vessels plays an important role in maintaining the viability of the tissue. Artificial blood vessels applied to the human body need to have various characteristics, such as elasticity, permeability, and biocompatibility comparable to the original vessels [60]. To control blood vessel fabrication, the printer should have sufficient resolution, and bioinks should not deform under the printing conditions [61]. In one study, treatment with angiogenin, a stimulator of angiogenesis, in a fibrin/bone powder scaffold enhanced angiogenesis and bone formation, compared to a control group [62]. Therefore, it is possible to add pro-angiogenic factors during 3D bioprinting to facilitate blood vessel formation in the 3D printed tissues.

### Application of 3D bioprinting technology for tissue regeneration

Recently, application of digital light processing stereolithography 3D printing technology for production of biodegradable polymeric vascular grafts has been reported [63]. Vascular grafts formed by 3D printing of human umbilical vein cells with poly propylene fumarate were applied for surgical grafting in patients with cardiovascular defects, suggesting that 3D bioprinting is highly useful for production of patient-specific vascular grafts [63]. In addition, 3D printing is also used for bone regeneration. Printed calcium phosphate scaffold have been widely used for bone regeneration [64]. Transplantation of calcium phosphate scaffold has proved effective in multiple animal studies [65]. Methods for increasing the osteogenicity of stem cells by applying polydopamine have also been developed [66]. In addition, 3D printing can be applied for cartilage regeneration. In one study, nanofibrillated cellulose plus alginate were used as scaffolds for making ears formed with a 3D printer, and the survival rate of chondrocytes in the scaffolds after transplantation was 73 to 86% [67]. In the case of bone and cartilage tissues, the size and shape of defects that occur in individual patients can be varied, therefore, 3D bioprinting technology may be highly useful for repair of damaged skeletal tissues [68].

Skin is the largest organ of the body, protecting the internal organs from external environments, retaining fluid, and acting as a sensory organ [69]. Thus, regeneration of skin wounds is important for not only cosmetic purposes but also restoration of physiologic function. In a clinical trial of treatment of burns, ulcers and other non-healing chronic wounds, stem cells have been proven to be an effective therapy for most patients [70]. In the case of burns or other large skin wounds, a method of transplanting through artificial skin fabricated out of polymers or human skin is widely used nowadays [71]. Although artificial skin substitutes for wound healing are commercially available, they have disadvantages such as a lack of viability, difficulty in reforming shape, and high costs [72]. It has been reported that skin-derived dECM bioinks can used to compensate for the rapid degradation and high contraction trends of traditional bioinks using conventional collagen. A printed mixture of adipose tissue-derived MSCs and endothelial progenitor cells with the skin-derived dECM for production of pre-vascularized skin grafts effectively accelerates cutaneous wound healing in animal models [73].

## Conclusions

Most therapies or treatments eventually aim to enhance tissue regeneration, and stem cell engineering has opened a new path to regenerative medicine. In this paper, we reviewed the current status of stem cell technologies, biomedical engineering, and nanotechnology for tissue regeneration. Biomedical engineering and nanotechnology will be helpful for overcoming the shortcomings of stem cell therapeutics by supporting stem cells to grow to an appropriate concentration, offering homogeneity, and resulting in proliferation at the desired location. However, biomaterials may cause toxicity when applied to the human body; hence, several methods have been developed to increase the biocompatibility of biomaterials. Tissue engineering can be applied for construction of various tissues, such as blood vessels, nervous tissue, skin, and bone. For stem cell engineering, several techniques should be developed involving new materials, new structures, and novel surface modifications of biomaterials; in addition, a deeper understanding of the interactions between cells and biomaterials will be needed.

## Abbreviations

BMPs: Bone morphogenic proteins
dECM: De-cellularized extracellular matrix
ECM: Extracellular matrix
MSCs: Mesenchymal stem cells
